# Preclinical Studies in Anti-*Trypanosomatidae* Drug Development

**DOI:** 10.3390/ph14070644

**Published:** 2021-07-05

**Authors:** Cintya Perdomo, Elena Aguilera, Ileana Corvo, Paula Faral-Tello, Elva Serna, Carlos Robello, Shane R. Wilkinson, Gloria Yaluff, Guzmán Alvarez

**Affiliations:** 1 Laboratorio de Moléculas Bioactivas, Departamento de Ciencias Biológicas, CENUR Litoral Norte, Universidad de la República, Paysandú 60000, Uruguay; cuquis266@gmail.com (C.P.); ilecorvo@gmail.com (I.C.); 2 Grupo de Química Orgánica Medicinal, Instituto de Química Biológica, Facultad de Ciencias, Universidad de la República, Montevideo 11400, Uruguay; elepao168@gmail.com; 3 Institut Pasteur de Montevideo, Unidad de Biología Molecular, Montevideo 11400, Uruguay; pfaral@pasteur.edu.uy (P.F.-T.); robello@pasteur.edu.uy (C.R.); 4 Departamento de Medicina Tropical, Instituto de Investigaciones en Ciencias de la Salud, Universidad Nacional de Asunción, San Lorenzo 2169, Paraguay; elvsern@hotmail.com (E.S.); gloriayaluff@yahoo.com (G.Y.); 5 Departamento de Bioquímica, Facultad de Medicina, Universidad de la República, Montevideo 11800, Uruguay; 6 Queen Mary Pre-Clinical Drug Discovery Group, School of Biological and Chemical Sciences, Queen Mary University of London, London E1 4NS, UK

**Keywords:** anti-trypanosomatid, arylidene ketones, thiazolidene hydrazines, Pathogen box, veterinary isolates

## Abstract

The trypanosomatid parasites *Trypanosoma brucei*, *Trypanosoma cruzi* and *Leishmania* are the causative agents of human African trypanosomiasis, Chagas Disease and Leishmaniasis, respectively. These infections primarily affect poor, rural communities in the developing world, and are responsible for trapping sufferers and their families in a disease/poverty cycle. The development of new chemotherapies is a priority given that existing drug treatments are problematic. In our search for novel anti-trypanosomatid agents, we assess the growth-inhibitory properties of >450 compounds from in-house and/or “Pathogen Box” (PBox) libraries against *L. infantum, L. amazonensis, L.* *braziliensis, T. cruzi* and *T. brucei* and evaluate the toxicities of the most promising agents towards murine macrophages. Screens using the in-house series identified 17 structures with activity against and selective toward *Leishmania*: Compounds displayed 50% inhibitory concentrations between 0.09 and 25 μM and had selectivity index values >10. For the PBox library, ~20% of chemicals exhibited anti-parasitic properties including five structures whose activity against *L. infantum* had not been reported before. These five compounds displayed no toxicity towards murine macrophages over the range tested with three being active in an in vivo murine model of the cutaneous disease, with 100% survival of infected animals. Additionally, the oral combination of three of them in the in vivo Chagas disease murine model demonstrated full control of the parasitemia. Interestingly, phenotyping revealed that the reference strain responds differently to the five PBox-derived chemicals relative to parasites isolated from a dog. Together, our data identified one drug candidate that displays activity against *Leishmania* and other Trypanosomatidae in vitro and in vivo, while exhibiting low toxicity to cultured mammalian cells and low in vivo acute toxicity.

## 1. Introduction

Neglected tropical diseases (NTD) represent a series of infections that cause illness in more than 1.7 billion people worldwide [1]. They are prevalent in low-income populations that live in developing areas of Latin America, Africa and Asia, and are responsible for approximately 150,000 deaths per year [2]. Additionally, it can limit the productivity in the workplace and make it difficult for the infected people to earn a living. It can impair the physical and cognitive development of children, and can lead to pathologies that result in social stigma. Together, these all contribute to trapping vulnerable individuals and their dependents in a disease/poverty cycle. Perversely, and for many NTDs, appropriate screening programs and cheap, simple to use prophylaxis and/or curative treatments are available but until recently the widespread implementation of such schemes has not been forthcoming. Driven by World Health Organization, Drugs for Neglected Diseases initiative, the Bill and Melinda Gates Foundation and other charity organizations, the true impact of NTDs on communities least able to deal with these major public health issues was brought to the attention of governments in the developed world and the pharmaceutical industry. This has promoted the implementation of prevention, control and treatment programs with the WHO launching a roadmap that aims to eliminate or eradicate 20 NTDs by 2030 [1].

Three NTDs, human African trypanosomiasis (HAT), Chagas disease and leishmaniasis, are caused by protozoa belonging to the family *Trypanosomatidae* [1]. The tsetse fly transmitted zoonotic infection, HAT is prevalent in 36 countries across sub-Saharan Africa and caused by subspecies of *Trypanosoma brucei*. In mammals, this parasite is found at extracellular sites being restricted in the early stages of infection to the host’s bloodstream and lymphatic system, where it causes relapsing fever, before eventually crossing the blood-brain barrier and gaining access to the cerebral spinal fluid, where it elicits diverse mental, neurological, sensory, and motor manifestations [3]. In contrast, Chagas disease is caused by *Trypanosoma cruzi*, an intracellular parasite that can invade any nucleated cell in any mammalian host. This zoonotic disease is transmitted by blood-sucking triatomine insects and endemic in 21 countries across Latin America. In the initial, acute stage of the disease, patients are generally asymptomatic although if symptoms are provoked, they tend to be non-specific, mild and flu-like (fever, fatigue, body aches, and headaches) [4]. As such, many Chagas disease cases generally remain undiagnosed [5] and in about 70% of situations, the infection does not progress any further. However, in the remaining instances, the disease can reactivate with the patient entering the chronic stage. Here, cardiac and gastrointestinal clinical manifestations arise leading to a multitude of heart-related conditions (arrythmias, conductive defects, cardiomegaly, etc.), megaesophagus and/or megacolon disorders that are invariably fatal [4]. Estimates indicate that up to 8 million people are currently infected with *T. cruzi* [1] and although incidence at endemic sites has decreased significantly over the last 20 years, the number of cases in non-endemic regions (United States, Australia, Europe, and Japan) driven by population migration, modern medical practices and congenital transfer, have increased: Estimates suggest that there are about 325,000 and 100,000 cases in the US and Europe, respectively [6], indicating that Chagas disease has truly emerged as a global infection.

The third NTD caused by a member of the Family Trypanosomatidae is leishmaniasis. This disease represents a group of sandfly transmitted zoonotic diseases caused by different *Leishmania* that dependent on the protozoal species, and host immune response affect the skin, mucosa, and/or viscera. As with *T. cruzi*, *Leishmania* are intracellular parasites but unlike their trypanosome counterpart, infect only macrophages and neutrophils. Infections are generally chronic, have low morbidity and moderate mortality, and based on the targeted area, can be disfiguring. Leishmaniasis is prevalent in 98 countries across 5 continents with an estimated 1.3 million new cases occurring each year. About one-third of new infections correspond to the life-threatening, visceral form of the disease, caused by *Leishmania donovani*, a human-to-human transmitted species that predominates in Africa and the Indian sub-continent, or *Leishmania infantum* (or its synonym *Leishmania chagasi*), a pathogen that can spread from animals (particularly canines) to humans and is found in the Mediterranean basin and Americas. These parasites promote widespread damage to the host’s internal organs that weaken the patient’s immune system and make them prone to secondary infections such as pneumonia, tuberculosis, dysentery or AIDS [7].

With no prospect of prophylaxis, curative chemotherapies currently represent the only way to treat Trypanosomatidae infections. However, their use can be problematic. For example, toxic and sometimes fatal side effects are encountered when using the difficult to dispense, arsenic-containing anti-HAT Melarsoprol, while alternative therapies based around Eflornithine and Nifurtimox may be expensive, require high dosages, have complex administration routes, are carcinogenic or have limited efficacy [8]). Likewise, drugs based on phosphocholine (Miltefosine), antimony (Glucantime and Pentostam), cyclic antibiotics (Amphotericin B), aminoglycosides (Paromomycin), and aromatic diamidines (Pentamidine) can be used against leishmaniasis but again there are concerns about the efficacy, cost, and adverse effects of such treatments [9]. To complicate this situation, strains that are non-responsive to treatment have emerged, limiting the usefulness of certain therapies in some regions [10,11,12].

Against this backdrop, there is an urgent need for new therapies targeting trypanosomal and leishmanial diseases, not only in humans, but also in animal reservoirs. Here, we evaluate the growth inhibitory properties of two chemical series, an in-house chemical collection and/or an open-access Pathogen Box (PBox) library, against Trypanosomatidae parasites.

## 2. Results and Discussion

The in-house chemical collection was tested against *L. infantum*, *L. amazonensis* and *T. brucei*, and outcomes compared with the previously reported anti-*T. cruzi* activities [13,14,15,16]: Data summarizing the in vitro and in vivo activity against *T. cruzi*, toxicology profiles, and potential mechanism of action of the four best hits arising from the previous studies are shown in Table 1. For the PBox library [17], we tested the growth inhibitory properties of the collection against a reference strain of *L. infantum* and an isolate obtained from the latest canine Leishmaniasis outbreak in the World [18,19]. We also collate information about commercial availability, literature reference citations, and other associated biological activity arising from promising hits following extensive literature reviews and database searches (PubChem, Scifinder^®^, Reaxys^®^, patents, etc). The hits identified here provide new chemical starting points for novel drugs targeting NTDs caused by Trypanosomatidae and could help individuals escape from the disease/poverty cycle.

### 2.1. Chemistry

The compounds used in this study can be made using simple one or two-step, environmentally friendly synthetic procedures (For thiazolidene hydrazine and arylidene ketone synthesis see Supplementary Information: Materials and Methods). The methods employed are readily scalable and facilitate the manufacture of small (grams) to large (kilograms) of each chemical and at low cost. Together, the chemistry involved in synthesizing chemicals from the in*-*house and PBox collections met several of the requirements expected when developing a new drug designed to target NTDs.

### 2.2. Antiparasitic Activity In Vitro

The growth inhibitory properties of more than 450 molecules derived from two chemical collections were tested against *Leishmania spp* or *T. brucei*. For the PBox library, approximately 20% of compounds were shown to have activity against *L. infantum* although variation in susceptibility phenotypes displayed by the two parasite strains tested were noted. For example, compounds A10, E2 and G7 displayed significant growth inhibitory activity (as judged by EC_50_ values of <15 μM) against the reference strain but were less effective towards the canine-derived isolate (EC_50_ values of >120 μM) while the reciprocal was observed when testing compounds B5, B11, C3 and H2 (Supplementary Figure S1). This variation in strain susceptibility is consistent with previous observations [19]. The underlying reason as to why the two *L. infantum* strains display different susceptibilities towards an array of unrelated chemotypes has yet to be established but given the adaptable nature of *Leishmania* we postulate that life cycle selection processes have helped shape the parasite’s genome leading to the strains with contrasting gene/protein expression profiles and the observed phenotypic variations [20,21,22]. This highlights that when screening for novel chemotherapeutics targeting a trypanosomatid parasite, multiple strains of the same species obtained from different sources should be tested.

Of those PBox library compounds that did display anti-*L. infantum* properties, 5 compounds (**MMV272144** [1-(3-Methoxyphenyl)-5-(methylsulfonyl)-1H-tetrazole], **MMV688761** [N-(1,3-Benzothiazol-2-yl)-4-methylsulfonyl-3-nitro-N-[[(2R)-oxolan-2-yl]methyl]benzamide], **MMV688768** [2-Methyl-3-[(R)-(4-methylpiperazin-1-yl)-thiophen-2-ylmethyl]-1H-indole], **MMV688763** [4-Chloro-5-{[5-(methylamino)-1,3,4-thiadiazol-2-yl]sulfanyl}-2-phenyl-2,3-dihydropyridazin-3-one] and **MMV021013** [N-Cyclohexyl-6-cyclopropyl-2-pyridin-2-ylpyrimidin-4-amine]) represented novel activities whose effect on this parasite had not been previously described, exhibiting equal potency towards the two strains tested (Table 2). Further phenotyping revealed these structures had no growth-inhibitory effect on murine macrophages over the concentration range tested (up to 50 μM) with most having selectivity index values (calculated as a ratio of the EC_50_ against the mammalian line to the EC_50_ against the parasite) equivalent or superior to Miltefosine (Table 2).

As these five compounds represent potential leads to treat visceral leishmaniasis, informatic searches were performed to explore the synthetic route for each chemical and evaluate for any synthetic difficulties and accessibility issue that may need to be addressed: Additional information above these five hits including structure/biological activities/LD50 values, are given in Supplementary Table S1. This indicated that **MMV272144** has a simple synthetic route but has a low in vivo absorption and low metabolic stability while **MMV688763** is reported to have pharmacokinetic problems (data provided by the DNDi program, non-published). In the case of **MMV021013**, its potential as an anti-leishmanial agent has previously been reported with it displaying significant activity against the *L. donovani* form found in a mammalian host [23,24]. Together with the observation that it can be cheaply synthesized and exhibits low cytotoxicity, **MMV021013** was proposed to be a good candidate for future in vivo studies [23,24].

Extending the screens to an in-house thiazolidene hydrazine series revealed that of the 20 compounds tested, 13 were deemed active (EC_50_ values < 20 μM) against the *L. infantum* reference strain, with **266**, **314** and **1147** also displaying growth inhibitory properties against the canine-derived isolate (Table 3): These three compounds represent the only structures to have activity against the veterinary isolate. To gauge how this library affects other trypanosomatids, their potency toward *L. amazonensis* and *T. brucei* was determined or, for *L.* *braziliensis* and *T. cruzi*, EC_50_ values sourced from published data [13,15,25]. This revealed that **266** and **314**, displayed growth inhibitory effects (EC_50_ values < 20 μM) against all the parasite species tested with their pharmacological profiles making them candidates for the treatment of leishmaniasis, Chagas disease and HAT. For **872**, activities against *T. cruzi*, *T. brucei* and three *Leishmania* isolates were observed (**872** displayed no growth inhibitory effect on the *L. infantum* canine-derived isolate) while **873**, **887**, **911**, **909**, **1115** and **1153** exhibited trypanocidal properties. Other compounds of note include **1102**, which in terms of its effect on *Leishmania,* behaved similarly to **872**, and **1147**, which affects both *L. infantum* strains and *L.* *braziliensis* but not *L. amazonensis*.

**Figure 1:**
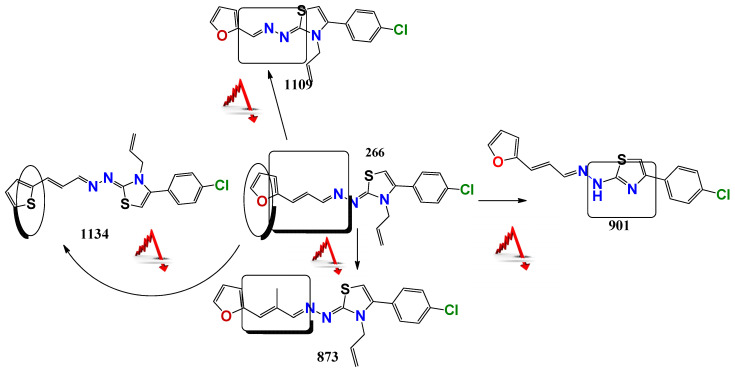
Structure-Activity Relationship a structure striping to decode the possible pharmacophore. This figure showing the changes in the antiparasitic activity related to little structure modification of the **266** compound. The red arrow minds a decrement in the antiparasitic activity.

For the thiazolidene hydrazine series a qualitative analysis of the structure-activity relationship was performed (Figure 1). We note that the resonance of electrons with the opening of the furan ring is a determinant of the biological activity in *Leishmania spp*., suggesting that this structural motif is part of the pharmacophore of the molecules. For example, comparing **1109,** which does not have a double bond conjugated to the furan ring, to **266**, only the latter has biological activity. If we vary the electron density throughout this conjugation by the incorporation of a methyl group (**873**), we observe that the activity decreases significantly in *Leishmania spp*., but not in *T. cruzi.* Comparing the furan-containing **266** with **1134,** its thiophene equivalent, a decrease in leishmancidal activity was observed possibly due to the lower probability of opening of the aromatic ring in thiophene compare to the furan ring [24]. This suggests a mechanism of action with an opened furan ring, as a toxic reactive biotransformed molecule.

Also, if we compare these molecules with compound **901**, the latter has better in vitro activity against *T. cruzi* but it has synthetic and biological activity problems because of tautomerization. The family of selenium compounds arises from the substitution of the sulfur atom by its bioisosteric selenium in the family of thiazoles described above. Four compounds were evaluated, of which **1147** was the one with the best biological activity, being their EC_50_ in *L. infantum (*ref and clinical isolate*)* of 5 and 9 µM, respectively. However, these compounds have more steps and synthetic difficulties, and lower stability than the parental ones.

In the case of 20 curcuminoid-based structures (Table 4), **799**, **795** and **796** were active against all the trypanosomatids strains tested with three others (**809**, **223** and **1019**) displaying anti-parasitic properties towards the *L. infantum, L. amazonensis*, *L.* *braziliensis* and *T. cruzi* reference strains: The three latter compounds showed no trypanosomicidal effect on the canine-derived *L. infantum* or *T. brucei*. From a pharmacological perspective, this family of compounds is often overlooked even though they do exhibit biological activities against a range of different organisms, including *Leishmania*, primarily because their classical structure is metabolically unstable [26].

We postulate that the metabolic stability of two of the best hits, **795** and **796**, may be increased as they contain furan or thiophene ring structures at the end of the carbon chain linker, respectively instead of phenolic groupings as found in classical curcuminoids. Even so, both structures are still considered Pan-Assay Interference Compounds (PAINS) because they also have groupings that can function as Michael acceptors with these able to nonspecific toxicity. However, despite extensive efforts, we were unable to identify what these nonspecific activities are for the compounds used here [14,27,28,29].

### 2.3. Toxicology

This section is divided into three parts: in vitro toxicity in mammalian cells and analysis of selectivity; genotoxicity by micronucleus test and acute oral toxicity in vivo. In Table 5 the nonspecific cytotoxicity of the compounds with the best antiparasitic activity was determined. For this, it was taken as a criterion that the compound has activity in the five species of parasites analyzed (*T. cruzi*, *T. brucei*, L *braziliensis*, *L. amazonensis*, and *L. infantum*) or that they are very active compounds in *L. infantum*. Compounds with a selectivity index greater than 10 were considered good and safe. This was performed by comparing the EC_50_ in macrophage cells (relevant cells in vivo infection with *L. infantum*) and the EC_50_ in vitro cultures of *L. infantum*, using the reference strain and the circulating isolate in Uruguay.

Of the 9 selected compounds with antiparasitic activity, 5 have selectivity indices greater than 10. However, all of them proved to be more selective than Glucantime, one of the reference drugs. Within the thiazolidene hydrazine family, of the two active hits, **314** was less selective than **266**. Furthermore, concerning the circulating strain in Uruguay, **314** was equally selective as Miltefosine, the drug used in the treatment of visceral Leishmaniasis [30], and was active in all the parasitic species analyzed. Within the curcuminoids family, we see that curcumin is not selective compared to the active derivatives **795**, **796,** and **799**. Despite being compounds that are usually considered PAIN, curcuminoids show that selective molecules can be found. Furthermore, compounds **796** and **799** showed biological activity in all the species of kinetoplastid studied. For all the compounds analyzed, the cytotoxicity towards fibroblasts was lower than for macrophages, which is another reason why the selectivity index was estimated using the latter cell type. Additionally, for compounds **796** and **314,** we analyze the cytotoxicity in human red blood cells and human macrophages at 50 µM and we did not see any inhibition of the cells grown. These results suggest a selective action against parasites and a large therapeutic ratio (more than 10 times) for these compounds.

In Table 6, the result of the micronucleus test is presented, used to evaluate genotoxicity with a treatment at a fixed dose in mice of 150 mg/kg of weight of compound **796**. A negative control is used to administer only the vehicle and positive control of intraperitoneal treatment with 40 mg/Kg of Cyclophosphamide, a known genotoxic agent. This assay is included in those recommended by the FDA to predict DNA toxicity during the drug development process. Furthermore, a prediction using the Toxicity Estimation Software Tool (TEST) for mutagenicity of **796** was negative. In previous works by our group, this test had already been carried out on compounds **266** and **314**, finding that they are not genotoxic. They also have a negative AMES test that evaluates the mutagenic capacity of the molecules [13,16].

Finally, the acute toxicity in mice of compound **796** was evaluated by the up and down test, resulting in an LD50 > 2000 mg/kg of weight. Hit compounds **266** and **314** were previously evaluated in our group with this test, obtaining the same result [13,16].

Table 7 shown the pharmacokinetic parameters that are commonly evaluated in the early stages of drug development, for the leading compounds and the reference drugs for Leishmaniasis and Chagas disease, Miltefosine and Benznidazole, respectively. All the evaluated compounds are poorly soluble in water (same as Miltefosine) and have solubility between 10 and 100 times less than Benznidazole. Likewise, Miltefosine and our compounds have high lipophilicity. On the other hand, the molecules studied show greater penetrability through the skin, a characteristic that may be advantageous for the topical administration of drugs for the treatment of cutaneous Leishmaniasis. Finally, it is interesting to note that the only compound that is predicted to cross the blood–brain barrier is hit **796**, which suggests that it could be active against *T. brucei* brain invasion and used for the treatment of sleeping sickness.

Because of the low stability of curcuminoid compounds, we performed a metabolic stability study with compound **796.** It was detected after 4 h without the emergence of any other metabolites measured under TLC analyses (see supporting information). The 4 h stability is one of the in vitro parameters recommended in the international drug development guidelines [31,32]. Compounds **799** and **795** were degraded before 1 h, so those compounds will not be considered further, due to their lesser metabolic stability.

### 2.4. In Vivo Proof of Concept

**Figure 2:**
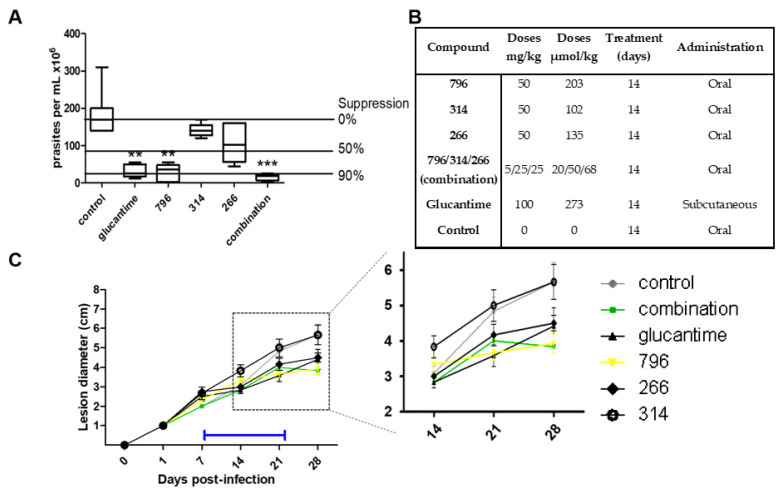
In vivo assay of cutaneous leishmaniasis. (**A**) Infections with *L. amazonensis* were established for 1 week in the right hind footpad of BALB/c mice.** and *** are statistically significant difference (0.1 and 0.05 respectively). Treatments of selected compounds were administered (see (**B**)) 1 week post-infection and maintained for 2 weeks. Animals were euthanized two weeks after cessation of treatment and the total parasite loads found in the lesion for the total of the animals for each treatment. (**B**) Details relating to the administration of each compound. (**C**) The right hind footpad of BALB/c mice was infected with *L. amanuensis*. Disease progression was monitored by measurement of the lesion diameter over a period of 28 days. Orally administered treatment of selected compounds (see key) was initiated 1 week post-infection and maintained for 2 weeks (blue bar). The insert represents an expanded image of lesion development from day 14 to 28 post-infection. The combination slope corresponds to an orally administered mixture of **266**, **314** and **796.**

Three compounds were selected to evaluate their in vivo efficacy in a murine model of cutaneous Leishmaniasis (**796, 314, 266**) and the results are presented Figure 2. This model was used for its simplicity and because it allows a significant reduction in the number of animals required for the test compared to the visceral mouse model of *L. infantum* in vivo infection. The visceral mouse model requires the sacrifice of the animals to follow the parasites throughout the treatment, getting in a cost of a large number of animals than the cutaneous model. Furthermore, we observed that in general, the active compounds behave similarly in the *Leishmania* species that cause skin and visceral disease studied in this work (*L. amazonensis* and *L. infantum*, respectively). To support this observation we perform a single dose study at 10 µM in the amastigote form of *L. infantum* for **796, 314, 266** and we found an inhibition % of 90, 85, and 69, respectively.

**Figure 3:**
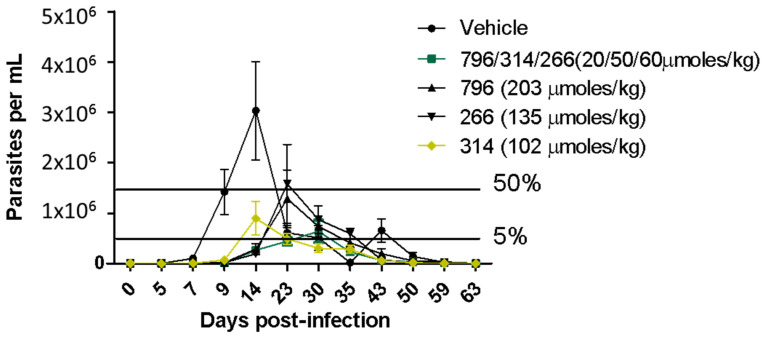
In vivo assay in the Chagas disease acute murine model. The oral administration was the same as in the in vivo experiment on Leishmania. The black bars indicate the 50 of parasitemia reduction with relation to the parasitemia peak in the vehicle control (group of infected animals treated only with the vehicle).

For the in vivo model of cutaneous disease, 1 × 10^7^ amastigote parasites of *L. amazonensis* PH8 were inoculated in the upper part of the left paw of the mice. As shown in Figure 3, on day 8 post-infection treatment was started for 14 days with the mentioned compounds and the reference drug, Glucantime. The mice were sacrificed 30 days post-infection. The antiparasitic effect was evaluated by two methods: the weekly evolution of the diameter of the infected paw throughout the experiment (Figure 2C) and the quantification of the parasite load in the infected area at the end of the experiment (Figure 2A).

Of the three leading compounds, **796** presented more than 50% suppression of parasites, behaving similar to the drug Glucantime but administered at a lower dose (1.3 times less). The % of suppression of the parasites for the other molecules was around 30%, but those molecules were administered at half of the dose of **796**. Regarding the mechanism of action, we perform experiments with Triosephosphate isomerase from *L. mexicana*, because compound **796** is analog to a potent triosephosphate isomerase inhibitor of *T. cruzi* [14], but this compound at 50 µM was not an inhibitor of this enzyme (see in supporting information). On the other hand, in Figure 2C we can see that when treatment with compounds **796** and **266** begins, the diameter of the paw begins to decrease, indicating a decrease in inflammation that is assumed to be directly proportional to the parasite load or at an anti-inflammatory effect.

We were surprised that low leishmanicidal activity in vivo was observed with the administration of compound **314**. This compound was the one with the best response in the in vivo model of infection with *T. cruzi* [16], reducing more than 60% of parasitemia with 100% survival (with a mean of 60% survival in the untreated group, Figure 3) at the same dose evaluated in the Leishmaniasis in vivo assay. This could be partly because we observed great variability in the final appearance of the formulations in the preparation of the vehicle with compound **314**, which we could not improve due to solubility problems. This is decisive in the correct absorption in vivo and the concomitant antiparasitic effect. Therefore, the observed variability between trials possibly reflects pharmacokinetic problems, since this compound would share the pharmacophore with hit **266**. Additionally, the dose of compound **314** was half that of **796**, and higher doses could be more effective.

Glucantime behaves similarly to our active compound **796**. When the drug administration is stopped, the diameter of the paw continues to increase, so the treatment time used was not enough to clear all the parasites. This shows that it is necessary to adjust the pharmacokinetic parameters and optimize the time and dose of administration of the compounds to obtain a full elimination of the infection. It should be noted that the anti-leishmanial effect of these compounds was obtained by oral treatment, which represents the preferred method of administration compared to the more invasive routes that require the use of injectable. Additionally, this oral administration gives information about the distribution, because the parasites are in the lesion at the dermic area, then the distribution seems to be systemic.

There is evidence that the co-administration of thiazolidene hydrazines and curcuminoids have synergistic effects on *T. cruzi* [33], so we decided to evaluate the three leading compounds at lower doses in the same formulation on the same murine leshmniasis model and also in the Chagas disease murine model (Figure 3). The dose of the most active compound **796** was reduced 10 times while the dose of hits **314** and **266** was reduced by half. In this experiment (Figure 2A), we observe higher parasite suppression than the monotherapy (**796** at 203 µmol/kg), and because the dose was 10 times less than monotherapy we conclude that there is a synergic effect between those compounds. We will perform isobolograms in vitro to find which combination and the optimal proportion between these compounds are producing the synergic effect. The trypanosomicidal activity in the murine model of Chagas disease was also synergic because the parasitemia was fully controlled by the reduced doses of this compound compared to the monotherapy (Figure 3).

## 3. Material and Methods

### 3.1. Cell Culturing

*L. amazonensis* and *L. infantum* (MHOM/BR/2002/LPC-RPV) were obtained from Fiocruz (Collection of Oswaldo Cruz Foundation, Rio de Janeiro, Brazil), while a *L. infantum* line (MCAN_UY_2015_gPL8) was isolated from a dog suffering from VCL [19]. *Leishmania* promastigotes were cultured at 28 °C in RPMI-1640 supplemented with glucose (0.7% (*w/v*)), ornithine (0.1% (*w/v*)), fructose (0.4% (*w/v*)), malate (0.6% (*w/v*)), fumarate (0.05% (*w/v*)), succinate (0.06% (*w/v*)), heat-inactivated fetal bovine serum (HI-FBS; 20% (*v/v*)), vitamins, and amino acids solution (Gibco, NY, USA). Once established, parasites in the exponential phase were transferred and cultured at 28 °C in an axenic medium consisting of BHI-Tryptose supplemented with FBS (10% (*v/v*)), hemin (2 × 10^−5^ mg/mL), glucose (0.03% (*w/v*)), streptomycin (2.0 × 10^−4^ g/mL), ampicillin (1.3 × 10^−4^ g/mL). *Leishmania* metacyclic parasites were harvested from promastigote cultures as described previously [18]. These were used to infect differentiated human acute monocytic leukemia (THP-1) cells at a ratio of 20 parasites per mammalian cell. The infected monolayers were incubated overnight at 37 °C under a 5% (*v/v*) CO_2_ atmosphere in a mammalian growth medium and then washed with RPMI-1640 to remove residual parasites. Leishmania amastigote parasites were maintained in differentiated THP-1 cells at 37 °C under a 5% (*v/v*) CO_2_ atmosphere in RPMI-1640 medium.

*T. brucei brucei* bloodstream form trypomastigotes (MITat 427 strain; clone 221a) were cultured at 37 °C under a 5% (*v/v*) CO_2_ atmosphere in modified Iscove’s medium supplemented with 10% (*v/v*) HI-FBS as described previously [34].

The J774.1 (ATCC^®^ TIB-67™) murine macrophage line was grown at 37 °C under a 5% (*v/v*) CO_2_ atmosphere in DMEM medium containing L-glutamine (4 mM) and HI-FBS (10% (*v/v*)) [16].

The THP-1 (ATCC^®^TIB-202) human acute monocytic leukemia line was grown at 37 °C under a 5% (*v/v*) CO_2_ atmosphere in RPMI-1640 medium containing 2-mercaptoethanol (50 μM) and HI-FBS (10% (*v/v*)). Differentiation of THP-1 to produce macrophage-like cells was carried out using phorbol 12-myristate-13-acetate (Sigma-Aldrich, Deisenhofen, Germany) [18].

### 3.2. In Vitro Antiparasitic Activity

All growth inhibition assays were performed in a 96-well plate format. *Leishmania* promastigotes (2 × 10^4^ cells) or *T. b. brucei* BSF trypomastigotes (2 × 10^3^ cells) were seeded in 200 μL growth medium containing different concentrations of the compound. The parasites were used at the exponential estate at the growing curve. Compounds were dissolved in dimethylsulfoxide (DMSO). After culturing the parasites at 28 °C for 2 days (*Leishmania*) or 37 °C under a 5% (*v/v*) CO_2_ atmosphere for 2 days (*T. b. brucei*), resazurin (9 μg for Leishmania or 2.5 μg for *T. b. brucei*) (Sigma Aldrich, Deisenhofen, Germany) was added to each culture and the plates incubated for a further 6–8 h. Cell densities were determined by monitoring the fluorescence of each culture using a Gemini Fluorescent Plate Reader (Molecular Devices, CA, USA) at an excitation wavelength of 530 nm, an emission wavelength of 585 nm and a filter cut off at 550 nm. The change in fluorescence resulting from the reduction of resazurin is proportional to the number of live cells. The effective concentration of a compound that inhibits cell growth by 50% (EC_50_) was established using OriginLab8.5^®^ sigmoidal. All assays were performed in triplicate on two experimental repeats.

Differentiated THP-1 monocytes (30,000 cells on an 18-mm round glass coverslip) were infected with *Leishmania* metacyclic parasites (30,000 parasites). Following incubation overnight at 37 °C in a 5% (*v/v*) CO_2_ atmosphere, the cultures were washed twice in a growth medium to remove noninternalized parasites and the supernatant was replaced with a fresh growth medium containing the compound under investigation. Compound-treated infections were incubated for a further 2 days at 37 °C under a 5% (*v/v*) CO_2_ atmosphere. Phosphate buffered saline-washed cells were fixed in 95% (*v/v*) ethanol, stained with 10% (*v/v*) Giemsa, visualized with Leica DMRA2 light microscope (Wetzlar, Germany) using a X100 oil immersion objective and images captured using a Retiga EXi Fast 1394 digital camera (Teledyne Imaging, AZ, USA). Infectivity was assessed by determining the number of infected cells and the number of parasites per infected cell. Infectivity index calculation = (% of infected cells × (amastigotes per cell))/number of total counted cells [18].

### 3.3. Nonspecific In Vitro Cytotoxicity in Mammalian Cells

Adhered J774.1 macrophages or differentiated THP-1 monocytes (1 × 10^4^ cells) were cultured in the compound-containing medium for 48 h to the compounds. Cell viability was then assessed using the 3-(4,5-dimethylthiazol-2-yl)-2,5-diphenyltetrazolium bromide (MTT) colorimetric assay [1]. In this analysis, MTT was added to a final concentration of 0.1 mg/mL to each well, and the culture was incubated at 37 °C for 3 h. The medium was removed, any formazan crystals dissolved in solubilization buffer (glycine (10 mM); NaCl (10 mM); EDTA pH10.5 (0.05 mM) in DMSO) and the absorbance at 560 nm determined. The effective compound concentration that inhibits cell growth by 50% (EC_50_) was established using OriginLab8.5^®^ sigmoidal. All assays were performed in triplicate [35].

To assess a compound’s hemolytic activity the Red Blood Cell Lysis Assay (national blood bank) was performed. A compound-containing erythrocyte (2% (*w/v*)) suspension prepared in phosphate-buffered saline pH 7.4 was incubated for 24 h at 37 °C and the amount of hemoglobin released into the supernatant determined spectrophotometrically using a Varioskan TM Flash Multimode Reader (Thermo ScientificTM, MA, USA) set to a wavelength of 405 nm. The % hemolysis was determined using the follows equation: % released hemoglobin = [(A_1_ − A_0_)/(A_1water_)] × 100, where A_1_ and A_0_ represent the absorbance at 405 nm of the test sample at 0 and 24 h, and A_1water_ the absorbance at 405 nm of water at 24 h. The experiments were performed by quintuplicate. Amphotericin B (final concentration of 1.5 µM) was used as a positive control [13].

### 3.4. Vehicles/Formulation Preparation

The compounds (at the doses described in the appropriate section) used for the in vivo assay were vehiculated in a mixture composed of a surfactant (10%) containing Eumulgin HRE 40 (polyoxyl-hydrogenated castor oil), sodium oleate, and soya phosphatidylcholine (8:6:3), and an oil phase (10%) containing cholesterol and PBS (80%). For formulation, cholesterol, Eumulgin HRE 40, and phosphatidylcholine previously pulverized in mortar were dissolved in chloroform and the solvent was evaporated under vacuum to dryness. In parallel, sodium oleate was dissolved in phosphate buffer and left in an orbital shaker for 12 h at room temperature. The latter was then added to the evaporated residue, and the mixture was homogenized and placed in an ultrasonic bath at full power for 30 min and kept at room temperature until use [13]. The volume used as a single dose on 0.2 mL, and then 30 mL of the compound at the respective doses were prepared. The doses were prepared according to the mouse weight at the time of the experiment.

### 3.5. In Vivo Micronucleus Test

For the in vivo micronucleus test, approximately three-month-old CD-1 male mice were housed in polycarbonate cages at room temperature (25 °C) and a photoperiod of 12 h throughout the study. The selected compound/vehicle was orally administered twice, at days one and two, to groups of five mice at a dose of 150 mg/kg of body weight. Mice were sacrificed 24 h after the last administration, and the bone marrow prepared for evaluation as described with slight modification [16]. At least two slides of the cell suspension per animal were made. The air-dried slides were stained with Giemsa stain (5% in phosphate buffer, pH 7.4) and examined at 1000× magnification. Small round or oval bodies, the size of which ranged from about 1/5 to 1/20 of the diameter of a polychromatic erythrocyte (PCE), were counted as micronuclei. A total of 1000 PCEs were scored per animal by the same observer for determining the frequencies of micronucleated polychromatic erythrocytes (MNPCEs). Cyclophosphamide (50 mg/kg) administered intraperitoneally (i.p.) 24 h before mouse sacrifice, was used as a positive control. For statistical analysis, the homogeneity of variances of data was tested by the analysis of variance (ANOVA) test (*p* < 0.05) using the EpiInfo (3.5.1) software.

### 3.6. In Vivo Acute Oral Toxicity in Mice

The in vivo 50% lethal dose (LD_50_) of selected compounds using healthy young adult male B6D2F1 mice (30 days old, 25 to 30 g) was determined according to the guidelines of the Organization for Economic Cooperation and Development (OECD). Initially, the compound was dissolved in the vehicle described above (see Section 2.4) and was administered at 2000 mg/kg, by orogastric cannula, to one animal. The animal was fasted, maintained, and observed for 48 h. If the mouse survived, another animal received the same dose, and 48 h later, a third animal. If there were no signs of toxicity, the experiment was halted 14 days after administration, with the euthanasia of the animals according to the OECD guidelines. Observations of the general status of the organs were performed after sacrifice. The PROTOX software was used to predict the LD_50_ of the compounds (http://tox.charite.de/protox_II/, accessed on 5 October 2018) [16]. The mutagenicity prediction was using the Toxicity Estimation Software Tool (TEST) [36].

### 3.7. In Vivo Anti-Leishmania Studies in Cutaneous Mice Model

Golden hamsters (*Mesocritus auratus*) were used to maintain *L. amazonensis* infections with the parasites passage every 6 to 8 weeks. BALB/c mice (female and male) supplied by the IFFA-CREDO, Lyon, France, and bred at the Instituto de Investigaciones en Ciencias de la Salud, Asuncion, Paraguay, were inoculated in the right hind footpad with 2 × 10^6^ *L. amazonensis* amastigotes in 100 µL PBS obtained from donor hamsters. Disease progression was monitored by the measurement of lesion diameters for 7 to 12 weeks. In all experiments, treatment was initiated 1 or 2 weeks after inoculation, when infection had become established and lesions obvious. Two days before the administration of the drug, the mice were randomly divided into groups of eight. *N*-Methylglucamine antimonate was administered subcutaneously to the BALB/c mice (100 mg/kg) for 20 days while the selected compound was administered orally at 50 mg/kg body weight. The animals were euthanized two weeks after cessation of treatments to assess parasitological loads in the infected footpad. Briefly, the mice were sacrificed, and the lesions of the infected footpad excised, weighed, and homogenized in a tissue glass grinder and the homogenate suspended in 1 mL RPMI-1640 (Gibco, NY, USA) supplemented with 10% (*v/v*) FBS, glutamine (29.4 µg/mL), penicillin (100 U/mL), and streptomycin (100 µg/mL). Following incubation at 27 °C for seven days, cultures were examined with an inverted microscope (Olympus, Tokyo, Japan) at a magnification of ×400. The number of parasites per gram in the lesion was calculated by the following equation: Parasite burden = geometric mean of the number of parasites in each duplicate/(number of microscope field counted × weight of lesion × (25,000) hemocytometer correction factor) [37]. The mean and standard deviations were calculated by using OriginPro9 and GraphPad Prism5. Comparisons of parasite suppression in the infected footpads of the untreated and drug-treated groups were performed by Student’s *t*-test. One-way ANOVA (as a non-parametric statistical test) was also used on ranks.

### 3.8. In Vivo Studies in the Acute Model of Chagas Disease in Mice

Three-month-old Balb/c mice (day 0) were infected with infected blood from mice at the beginning of the peak of parasitemia (parasitemia greater than 1.0 × 10^6^ p/mL), with the CL Brener clone of T. cruzi (10,000 parasites per mouse), was inoculated intraperitoneally. Parasitemia was followed from the fourth day post-infection and until all the mice in the group were positive. Parasitemia measurements were made by the Hemoconcentration and counting micro method. Once all the mice were positive, the treatment was started. The treatment lasts 15 consecutive days, the compounds were administered orally by intragastric cannula once a day. Parasitemia was monitored weekly, at 30 and 60 days 200 μL of blood was extracted from the mouse tail for serological tests (ELISA test to detect antigens of *T. cruzi*). Day 60 was the end of the experiment and the animals were sacrificed. Hemoconcentration and counting micro method; the mice were bled by pricking the tail and taking the blood with capillaries. One capillary per mouse draws 8–18 mm in height of blood [37]. The millimeters were converted to μL by a table previously described and calibrated in the laboratory. The capillaries were centrifuged at 3000× *g* for 40 s. The parasitic load in the capillary was observed by optical microscopy (OM) in the capillary the Red Blood Cells (GR)) were distributed at one end and the supernatant serum, at the interface are the trypomastigotes. Then the capillary was cut a few mm on the interface towards the GR part were spread on a slide, covered with a coverslip and the parasites were counted in 50 fields (OM at 40× magnification), the total number of microscopic fields was calculated and the factor corresponding to 1/50 of this total number of fields. This factor, multiplied by the number of trypomastigotes counted in each slide, gives us the number of trypanosomes per 5 mm^3^ and is finally compared with the figures of the control animals. For serology, they were taken in the same way 4 capillaries filled with blood were cut and stored in tubes to perform the ELISA. The mean and standard deviation were calculated by using OriginPro9 and GraphPadprisma5. Comparisons of parasite suppression were performed by the analysis of variance (two-way ANOVA as a non-parametric statistical test).

### 3.9. Liver Fraction Stability Studies and Calculation of Pharmacokinetic Parameters

For the determination of the stability in the different fractions, (cytosolic and microsomal of rat hepatocytes) the different proteins present in them were used. The fractions were prepared according to the previously reported protocol [38]. The protein concentration in the different fractions was determined by the Sigma bicinchoninic acid (BCA) assay, as suggested in the manual. The final concentration of the molecules in the aqueous medium was 400 µM and prepared from a stock in DMSO of 40 mM. The solutions were homogenized and incubated at 37 °C at 10 min, 30 min, 1 h, 2 h, 3 h and 4 h. (by TLC). For this, it was incubated at 37 °C in a volume of 1 mL: 2.5 µL of 30 mM Magnesium Chloride (MgCl); 2.5 µL of 40 mM Nicotinamide Adenosine Dinucleotide Phosphate (NADP+); 5 µL of 350 mM Glucose 6- Phosphate (Glu6P); 5 µL of Glucose 6- phosphate dehydrogenase (Glu6PD) 50 U/mL, 5 µL of the stock of 40 mM compounds and the volume of the Phosphate Buffer (pH = 7) must be such that cytosolic (CF) and microsomal (MF) fraction FC and FM present a final protein concentration of 0.1 mg/mL. After that, the stability of compounds **796** and **1019** in the cytosolic (FCF) and microsomal (FMF) fraction of rat hepatocytes was evaluated at different times (1–4 h). Thin-layer chromatography of ethyl acetate extracts was conducted to evaluate the presence of decomposition products. The mobile phase used for these TLCs was n-hexane: ethyl acetate (7: 3).

Predictions were made using the open-access SwissADME software (http://www.swissadme.ch accessed on 5 of October 2018), a tool that allows the prediction of different pharmacokinetic parameters such as water solubility, gastrointestinal absorption, skin penetrability, lipophilicity, bioavailability, etc. [39] (in supporting information). The SwissADME software input uses the SMILES codes of the molecules, which were generated with the ChemBioOffice 2010 program.

## 4. Conclusions

We identified five drug candidates for in vivo studies in the visceral Leishmaniasis model from PBox. These candidates can be used as inspiration for new and potent redesigned molecules. We suggested a new function or tool for the drug collection such as PBox in the molecular characterization of a new cell strain. Additionally, we showed a lack of representation of reference strains of *L. infantum* in the drug discovery process.

Of the 50 compounds evaluated from our chemical collection, we conclude that three of the leading compounds (**796**, **266**, **314**), were found to have good antiparasitic activity in different species of trypanosomatids. Furthermore, these molecules have low nonspecific cytotoxic effects and did not show genotoxic or mutagenic effects. In addition to these encouraging results, they are safe, showing 100% survival in the acute toxicity model in vivo. None of the 400 compounds from the PBox have multi antiparasitic activity against *T. cruzi*, *T. brucei*, *L. amazonensis*, *L. braziliensis,* and *L. infantum* as our chemical collection has.

Regarding the mechanism of action of compound **796**, it should be noted that triosephosphate isomerase (TIM) does not appear to be the molecular target of the compound, regarding the low inhibition effect showed in *Lm*TIM and *Tb*TIM at 42.5% and 61% after treatment with 50 µM (in supporting information). Therefore this matter remains elusive and more studies are needed to assess this. Concerning the pharmacokinetic parameters of the compounds that were tested in the in vivo model, it is observed that the predictions were similar to the parameters shown by Miltefosine. Some are even estimated to have higher levels of gastrointestinal absorption and skin absorption, desirable qualities for a drug to be used in the treatment of Leishmaniasis.

It is important to mention that the oral administration of the compounds used in the in vivo model has many advantages in this type of disease, where the cost of the drug must be low and its route of administration simple. Additionally, the treatment has to be suitable for use in dogs.

Compound **796** was the most promising compound with effective control of the Leishmania parasite infection in vivo. Additionally, the synergic effect was observed between the two chemical groups (curcuminoids and thiazolidene hydrazines). Taken together, the results obtained encourage us to continue with the clinical phase of experimentation in the canine Leishmaniasis model of our leading compounds.

## Figures and Tables

**Table 1:** Anti-*T. cruzi* activity and general toxicological profile of the four best hits identified from our in-house chemical collection ^a^.

Compound ^a^			
314	1019	796	266
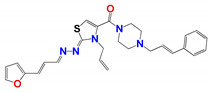	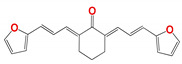	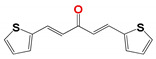	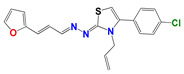
**Activity in vitro anti-*T. cruzi* (multiple strains)**			
^b^EC_50_ 0.72 µM Amastigotes	EC_50_ 0.60 µM epimastigotes	EC_50_ 5.0 µM epimastigotes	EC_50_ > 0.25 µM amastigotes
Selectivity index > 100 (EC_50_ mammalian cell/EC_50_ *T. cruzi*)			
**Mechanism of action**			
Cruzipain ^c^IC_50_ 4.3 µM	triosephosphate isomerase IC_50_ 86 nM	unknown	unknown
**Stability in vitro (microsomal, plasma, other solutions)**			
High	low	moderate	high
**Toxicology and Efficacy**			
**Ames Test (mutagenicity)**			
No	no	unknown	no
**Micronucleus test in mice (Genotoxicity)**			
No	unknown	unknown	no
**Acute oral toxicity (up and down test)**			
^d^ LD_50_ > 2000 mg/kg in mice	unknown	unknown	LD_50_ > 2000 mg/kg in mice
Full control of the parasitemia in vivo at 50 mg/kg in the murine model of Chagas disease			

^a^ Data were taken from [13,14,15]. ^b^ EC_50_ refers to the effective concentration of compound that inhibits cell growth by 50% ^c^ IC_50_ the concentration of compound required to inhibit enzyme activity by 50%, ^d^ LD_50_ the concentration of a compound that kills 50% of the animals tested in the in vivo acute oral toxicity assay.

**Table 2:** Leishmanicidal and selectivity properties of five novel hits identified from the PBox library.

Compounds	EC_50_ (μM)			Selectivity Index ^b^	
	*L. infantum* ^a^		MΦ Cytotoxicity ^a^	MΦ/Reference	MΦ/Veterinary
	Reference	Veterinary			
**MMV272144**	2.4 ± 0.3	1.2 ± 0.1	>50	>21	>42
**MMV688761**	4.9 ± 0.1	3.9 ± 0.3	>50	>10	>13
**MMV688768**	9.8 ± 0.5	6.8 ± 0.1	>50	>5	>7
**MMV688763**	2.1 ± 0.2	0.9 ± 0.1	>50	>24	>56
**MMV021013**	0.4 ± 0.1	0.3 ± 0.1	>50	>125	>167
**Miltefosine ^c^**	5.3 ± 0.1	4.1 ± 0.1	50 ± 7	10	12

^a^ Compounds were screened against reference (MHOM/BR/2002/LPC-RPV) and veterinary isolate (MCAN_UY_2015_gPL8) strains of *L. infantum* while mammalian cytotoxicity assessed using J774.1 murine macrophages (MΦ). The *Leishmania* EC_50_ values are means ± standard deviation from assays performed in triplicate. ^b^ The selectivity index corresponds to the fold difference in EC_50_ values of J774.1 murine macrophages versus the reference or veterinary isolate *L. infantum* strains. ^c^ For comparative purposes, the *L. infantum* EC_50_ data for Miltefosine were taken from [19].

**Table 3:** Susceptibility of Trypanosomatidae parasites to thiazolidene hydrazines.

Chemical Code	EC_50_ ± SD (μM) ^a;^^b^					
	*Lin–Ref*	*Lin–Vet*	*L. amaz*	*L. brazils* ^c^	*T. cruzi* ^c^	*T. brucei*
**Nifurtimox ^d^**	6.0 ± 1.0	10.0 ± 2.0		6.0 ± 2.0	7.0 ± 2.0	1.44.0 ^e^
**Glucantime ^d^**	26.0 ± 1.0		18.0 ± 2.0	20.0 ± 9.0		
**Miltefosine ^d^**	5.3 ± 0.1 ^f^	4.1 ± 0.1 ^f^		5 ± 2	8 ± 2	
**1385**	9.0 ± 1.0				19.0 ± 3.0	
**1109**						
**266**	2.0 ± 0.2	9.0 ± 1.0	7.0 ± 1.0	20.0 ± 2.0	1.6 ± 0.5	5.0 ± 1.0
**872**	10.0 ± 5.0		10.0 ± 1.0	8.0 ± 2.0	3.0 ± 0.5	6.0 ± 1.0
**873**					0.09 ± 0.02	14.0 ± 3.0
**1153**					<3.0	6.0 ± 1.0
**295**					3.5 ± 0.2	
**133**						
**877**					15 ± 3	10 ± 1
**1134**						
**314**	1.3 ± 0.5	2.5 ± 1.0	12.0 ± 5.0	4.0 ± 1.0	3.1 ± 0.2	5.0 ± 1.0
**1112**	1.5 ± 0.2				1.2 ± 0.2	12.0 ± 3.0
**1115**					11.0 ± 4.0	7.0 ± 2.0
**901**					1.2 ± 0.3	5.0 ± 1.0
**1119**	<6.0				10.0 ± 2.0	12.0 ± 1.0
**1102**	5.0 ± 2.0		16.0 ± 4.0	14.0 ± 2.0	1.6 ± 0.3	14.0 ± 2.0
**1140**						
**912**	<0.4		18.0 ± 5.0			
**903**	3.0 ± 1.0				12.0 ± 2.0	17.0 ± 5.0
**263**	<0.3				5.0 ± 1.0	
**909**	10.0				6.0 ± 1.0	19.0 ± 1.0
**1366**					12.0 ± 2.0	
**1367**						
**1369**	8.0 ± 2.0				15.0 ± 5.0	
**1222**						
**1219**						
**1147**	5.0 ± 1.0	9.0 ± 2.0		<12.0		
**1097**						

^a^ Growth inhibitory effect as judged by mean EC_50_ ± standard deviation of thiazolidene hydrazines (structures in Figure S3) on *L. infantum* reference (*Linf–ref*) and veterinary (*Lin–vet*) strains, *L. amazonensis (L. amaz)*, *L.* *braziliensis (L. brazils), T. cruzi* or *T. brucei*. ^b^ Green boxes represent compounds with anti-parasitic activity (EC_50_ values < 20 μM) with the EC_50_ value indicated. Red boxes signify compounds with no anti-parasitic activity (EC_50_ values > 20 μM). Clear boxes represent compounds whose activity was not determined. ^d^ control drugs ^c,e,f^ Data from [13,16,19,23,25] respectively.

**Table 4:** Susceptibility of Trypanosomatidae parasites to curcuminoids.

Chemical Code	EC_50_ ± SD (μM) ^a;^^b^					
	*Lin–Ref*	*Lin–Vet*	*L. amaz*	*L. brazils* ^c^	*T. cruzi* ^c^	*T. brucei*
**Glucantime ^d^**	26.0 ± 1.0		18.0 ± 2.0	20.0 ± 9.0		
**Curcumin**	5.0 ± 1.0			6.0 ± 1.0	6.0 ± 1.0	8.0 ± 1.0
**797**					11.0 ± 2.0	
**799**	<0.3	12.0 ± 3.0	5.0 ± 1.0	4.2 ± 0.9	5.0 ± 1.0	1.7 ± 0.5
**800**					14.0	15.0 ± 1.0
**795**	5.0 ± 1.0	10.0 ± 2.0	10.0 ± 6.0	6.0 ± 2.0	24.0 ± 2.0	1.8 ± 0.5
**793**	<0.3				5.0 ± 0.7	17.0 ± 2.0
**809**	3.0 ± 1.0		16.0 ± 2.0	6.0 ± 1.0	8.0 ± 2.0	
**1223**	<0.3		18.0 ± 4.0	16.0 ± 4.0	5.0 ± 2.0	
**1019**	<0.3		7.0 ± 1.0	13.0 ± 7.0	0.6 ± 0.2	
**1282**						
**796**	4.0 ± 0.5	10.0 ± 2.0	8.0 ± 2.0	4.0 ± 0.5	5.0 ± 0.8	0.6 ± 0.1
**1387**					16.0 ± 2.0	0.7 ± 0.1
**1414**					12.0 ± 1.0	16.0
**798**	3.0 ± 0.5			19.0 ± 5.0	13 ± 1	
**1018**	<0.3			11.0 ± 3.0	0.04 ± 0.01	15.0 ± 2.0
**1245**	<0.3			16.0 ± 1.0	0.6 ± 0.2	16.0 ± 3.0

^a^ Growth inhibitory effect as judged by average EC_50_ values ± standard deviation of thiazolidene hydrazines (structures in Figure S4) on *L. infantum* reference strain (*Linf–ref*), *L. infantum* veterinary isolate (*Lin–vet*), *L. amazonensis (L. amaz)*, *L.* *braziliensis (L. brazils), T. cruzi* or *T. brucei*. ^b^ Green boxes represent compounds with anti-parasitic activity (EC_50_ values < 20 μM) with the determined EC_50_ value shown. Red boxes signify compounds with no anti-parasitic activity (EC_50_ values > 20 μM). Clear boxes and nd represent compounds whose activity was not determined. ^c^ Data from [14] ^d^ control drug.

**Table 5:** Cytotoxicity and selectivity index of reference drugs and selected molecules.

Chemical Code	EC_50_ ± SD (µM) ^a^		Selectivity Index ^b^		
	MΦ ^f^	Fibroblasts	MΦ/*L. amaz*	MΦ/*Lin–Ref*	MΦ/*Lin–Vet*
**Nifurtimox ^c^**	200 ± 9	nd	33	33	20
**Glucantime ^c^**	15 ± 1	nd	0.83	0.57	Nd
**Miltefosine ^c^**	50 ± 7	nd	10	56	10
**266 ^d^**	60 ± 6	405 ± 10	9	30	7
**872 ^d^**	66 ± 7	319 ± 16	7	8	2
**314 ^d^**	30 ± 5	346 ± 9	3	23	12
**Curcumin ^e^**	10 ± 2	nd	2	2	Nd
**795 ^e^**	115 ± 2	114 ± 6	11	23	11
**809 ^e^**	33 ± 8	543 ± 6	2	6	Nd
**796 ^e^**	38 ± 7	158 ± 5	5	10	4
**799 ^e^**	115 ± 8	nd	23	>63	10

^a^ Growth inhibitory effect as judged by average EC_50_ values ± standard deviation against murine macrophages (MΦ) and fibroblasts (NCTC929). nd represents not determined. ^b^ selectivity index represents a ratio of the EC50 value against the mammalian cell line/EC50 against the *L. infantum* reference strain (*Linf–ref*), *L. infantum* veterinary isolate (*Lin–vet*) or *L. amazonensis (L. amaz)* (parasite data in Table 3 and Table 4). nd represents not determined. ^c^ control drugs. ^d,e^ thiazolidene hydrazine or curcuminoid-based structures, respectively. ^f^ MΦJ774.1 macrophages or differentiated THP-1 monocytes.

**Table 6:** Micronucleus test for compound **796** (150 mg/kg).

Treatment ^a^	Number EPMn ^b^	Number EPC ^c^	Media Mn/Mouse ± SD ^d^
**Control**	19	5000	4 ± 1
**796**	21	5000	5 ± 1
**Cyclophosphamide**	180	5000	36 ± 2

^a^ Five identical assays are performed at independent times. ^b^ Total polychromatic micronucleated erythrocytes (EPMn) found in the 5 assays. ^c^ Polychromatic erythrocytes (EPC) were observed in total. ^d^ Percentage of polychromatic micronucleated erythrocytes (EPMn) ± standard deviation.

**Table 7:** Prediction of pharmacokinetic parameters using SwissADME software.

Compound	Solubility (mg/mL)	Gastrointestinal Absorption	BBB Permeability	Penetrability of Skin (cm/s)	Bioavailability	Lipophilicity (LogP)
**Miltefosine**	1.9 × 10^−3^	Low	no	−4.0	0.55	3.8
**Benznidazole**	2.3	High	no	−7.2	0.55	0.5
**314**	3.5 × 10^−3^	High	no	−6.3	0.55	4.2
**266**	2.2 × 10^−3^	High	no	−5.2	0.55	4.8
**796**	3.9 × 10^−2^	High	yes	−5.3	0.55	3.7

## Data Availability

Data is contained within the article.

## Supplementary Materials

The following are available online at https://www.mdpi.com/article/10.3390/ph14070644/s1, Figure S1. Susceptibility of *L. infantum* towards compounds in the PBox library, Table S1. General activity profiles previously reported of the five hits identified in the phenotypic screening, Table S2. Compounds structures and compound preparation and characterization, Table S3. Activity of compound 796 in Triosephosphate isomerase from *L. mexicana* (LmTIM) and *T. brucei* (TbTIM).
