# Supplementary material for: Preclinical Studies in Anti-Trypanosomatidae Drug Development
Source: Pharmaceuticals (Basel). 2021 Jul 5;14(7):644. doi: 10.3390/ph14070644 (PMC8308625; doi:10.3390/ph14070644)
Supplement: Supplementary file 1 [file pharmaceuticals-14-00644-s001.zip › pharmaceuticals-1265240-supplementary.pdf]

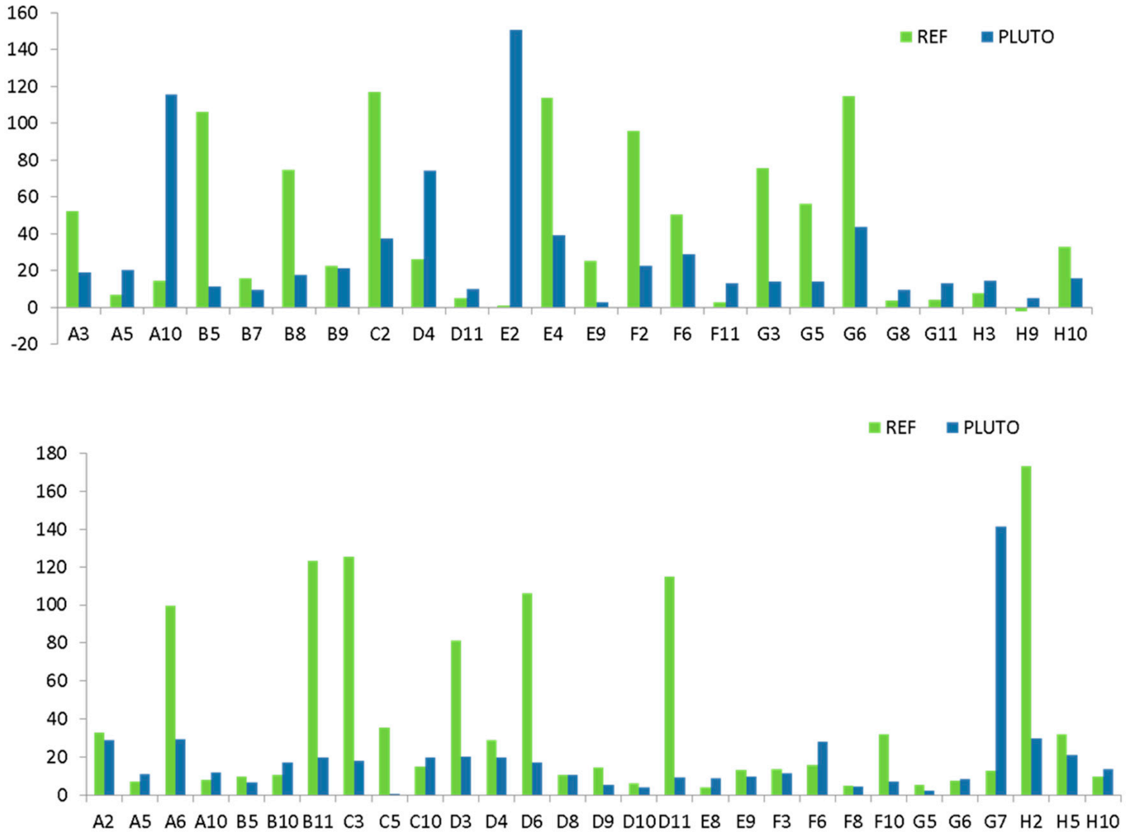

Supplementary Figure S1. Susceptibility of *L. infantum* towards compounds in the *PBox* library. The growth inhibitory properties of compounds from the *PBox* library were assessed against a *L. infantum* reference strain (green bars) and a line isolated from dogs PLUTO (blue bars). The y axes correspond to grow parasites %, and the x axes is the location in the well plates from the pathogen box plates.

Table S1. General activity profiles previously reported of the five hits identified in the phenotypic screening.

EC_{50} (μM)[a]	Target (previously reported biological activities)
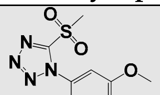 **MMV272144**	
15	Cell division cycle 42 (GTP binding protein, 25kDa) [*Homo sapiens*]
10	Neuropeptide S receptor isoform A [*Homo sapiens*]
12.5	Aldehyde dehydrogenase 1 family, member A1 [*Homo sapiens*]
1.3	Sphingosine-1-phosphate receptor 4 [*Homo sapiens*]
1.8	Fluorescence Cell-Based Retest of C. albicans Growth in the Presence of Fluconazole
7.3/1.1	Recombinase A [*Mycobacterium tuberculosis* H37Rv]
6.6	Hsf1 protein [*Mus musculus*]
3.1	Replicative DNA helicase [*Mycobacterium tuberculosis* H37Rv]
1750 mg/kg	Predicted LD50 in rats[b]
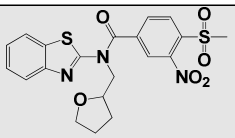 **MMV688761**	
11	*T. b. brucei* (Veale and Hoppe, 2018)
1000 mg/kg	Predicted LD50 in rats[b]
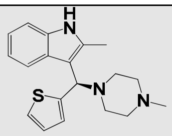 **MMV688768**	
3.1/32	*Candida albicans* Biofilm Inhibitor (Vila and Lopez-Ribot, 2017)/Human 535 hepatocellular carcinoma (HepG2) cell line.
1.5/4.7	*T. brucei brucei* /*P. falciparum* ABS activity (Duffy et al., 2017)
400 mg/kg	Predicted LD50 in rats[b]
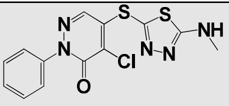 **MMV688763**	
2520 mg/kg	Predicted LD50 in rats[b]
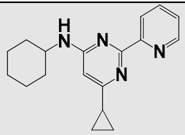 **MMV021013**	
0.8/400	*Leishmania donovani* (amastigotes)/HepG2 human cell line (Peña et al., 2015)
0.7	*P. falciparum* (Ballell et al., 2013)
1.7/3.5	*T. cruzi*/*T. brucei brucei* (Duffy et al., 2017)
300 mg/kg	Predicted LD50 in rats[b]

[a]EC_{50} is the effective concentration that cause a 50% of effects (cell grow inhibition or enzymatic inhibition). Data was taken from pubchem.ncbi.nlm.nih.gov, and verified using Scifinder® and Reaxys®. [b]PROTOX software was used to predict the lethal doses in rodents LD50 (http://tox.charite.de/protox_II/)

Table S2. *Compounds structures* [21,45].

THIAZOLIDENE HYDRAZINES		CURCUMINOIDS	
Structures	Chemical collection code	Structures	Chemical collection code
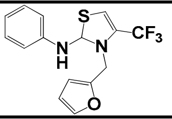	**1385**	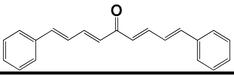	**797**
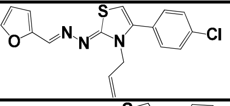	**1109**	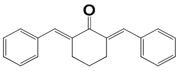	**799**
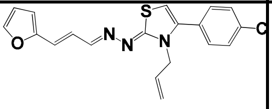	**266**	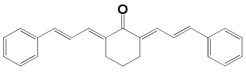	**800**
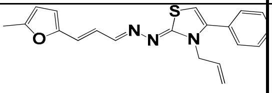	**872**	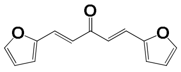	**795**
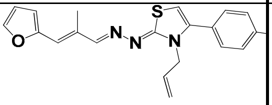	**873**	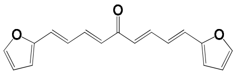	**793**
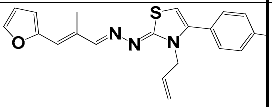	**1153**	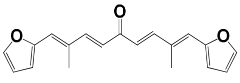	**809**
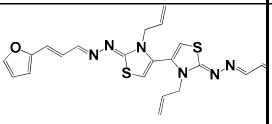	**295**	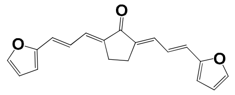	**1223**
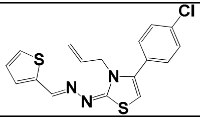	**133**	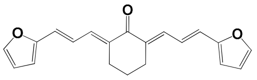	**1019**
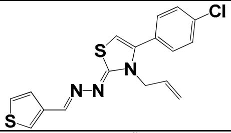	**877**	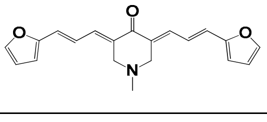	**1282**
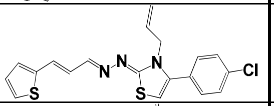	**1134**	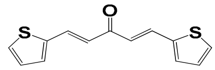	**796**
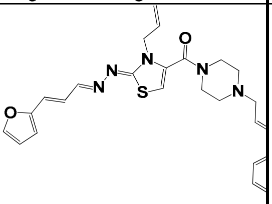	**314**	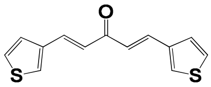	**1387**
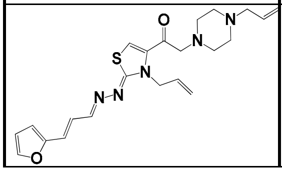	**1112**	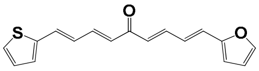	**1414**

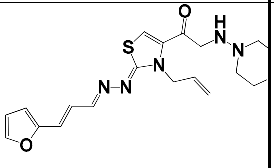	**1115**	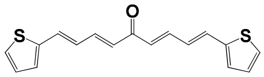	**798**
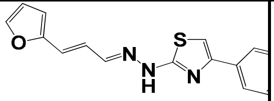	**901**	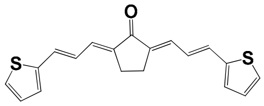	**1018**
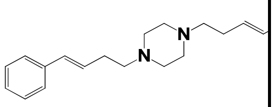	**1119**	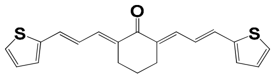	**1245**
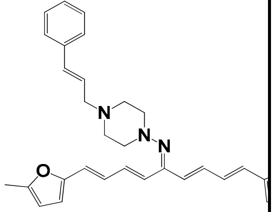	**1102**		
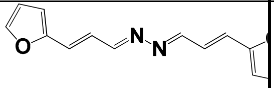	**1140**		
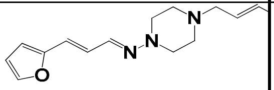	**912**		
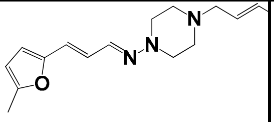	**903**		
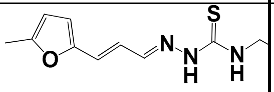	**263**		
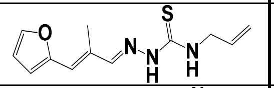	**909**		
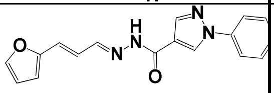	**1366**		
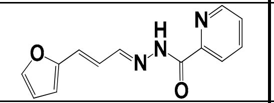	**1367**		
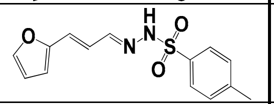	**1369**		
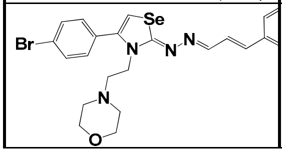	**1222**		

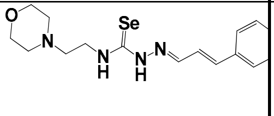	**1219**		
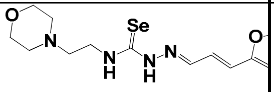	**1147**		
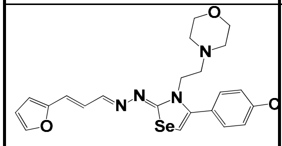	**1097**	Nd	Nd

Supplementary Information: Materials and Methods

2.1. Chemistry General

Reagents were purchased from Sigma-Aldrich and used without further purification. Melting points were performed using an Electrothermal Engineering Ltd. melting point apparatus, and the results were not corrected. 1H NMR and 13C NMR spectra were recorded in the indicated solvent with a Bruker DPX 400-MHz spectrometer. All solvents were dried and distilled before use. Reactions were monitored by thin-layer chromatography (TLC) using commercially available pre-coated plates (Merck Kieselgel 60 F254 silica), and the developed plates were examined under UV light (254 nm) or as iodine vapor stains. Column chromatography was performed using a 200-mesh silica gel. Mass spectrometry experiments were performed on a HEWLETT PACKARD MSD 5973 or an LC/MSD-Serie 100 using electronic impact (EI) or electrospray ionization (ESI), respectively. To determine the purity of the compounds, microanalyses were done on a Fisons EA1108 CHNS–O instrument from vacuum-dried samples and were within ± 0.4 of the values obtained by calculating their compositions. The compounds were prepared following synthetic procedures previously reported (Elena Aguilera et al., 2016; Alvarez et al., 2014; Álvarez et al., 2015).

2.1.1 General synthetic procedures of thiazolidene hydrazines (Álvarez et al., 2014).

A mixture of the corresponding aldehyde (1.05 equiv), the corresponding thiosemicarbazides (1.00 equiv), a catalytic amount of *p*-toluenesulfonic acid, and ethanol 98% (1 mL per 100 mg of aldehyde) was stirred at room temperature until the disappearance of the aldehyde (12–24 h, checked by TLC, SiO_2, and petroleum ether/EtOAc 70:30). After that, the precipitate was filtered off and washed with petroleum ether. The solid was crystallized from ethanol.

A mixture of the corresponding thiosemicarbazides (1.0 equiv), ethyl bromoacetate (2.0 equiv), anhydrous sodium acetate (4.0 equiv), and dry ethanol (1 mL per 100 mg of thiosemicarbazide) was heated at reflux until the disappearance of the thiosemicarbazide (4–10 h, checked by TLC, SiO_2, and petroleum ether/EtOAc 70:30). After that, the mixture was cooled at room temperature, and the precipitate was filtered off and washed with ethanol/water (80:20). The solid was crystallized from ethanol or ethanol/water.

A mixture of the corresponding thiosemicarbazide (1.0 equiv), the α -haloketone (2.0 equiv), and dry ethanol (1 mL per 100 mg of thiosemicarbazide) was heated at reflux until the disappearance of the thiosemicarbazide (4 to10 h, checked by TLC, SiO_2, and petroleum ether/EtOAc 70:30). After that, the mixture was cooled at room temperature, and the precipitate was filtered off and washed with ethanol/water (80:20). The solid was crystallized from ethanol or ethanol/water.

2.1.2 General synthetic procedures of arylidene ketones (Álvarez et al., 2017).

The corresponding ketone (0.316 g, 1.0 equiv) in ethanol (10 mL) was added to a concentrated aqueous solution of KOH (2.0 eq.). Then, the corresponding aldehyde (1.2 equiv) was added into the reaction mixture to get the corresponding benzylidine derivative. After completion, as revealed by the thin-layer chromatography (TLC) in an average span of around 1 h, the reaction mixture was precipitated using water because of the limited solubility. The precipitate was filtered, dried and purity was monitored through TLC. It revealed just a single spot which proved the presence of a single product. For further purification, the product was recrystallized from EtOH to obtain it as a solid

Table S3. Activity of compound 796 in Triosephosphate isomerase from *L. mexicana* (LmTIM) and *T. brucei* (TbTIM).

Expression and purification of proteins: TbTIM, LmTIM were expressed in Escherichia coli and purified as described in the literature [39]. After purification, the enzyme, disolved in 100 mM triethanolamine, 10 mM EDTA and 1 mM dithiothreitol (pH 8), was precipitated with ammonium sulfate (75% saturation) and stored at 4 _C. Before use, extensive dialysis against 100 mM triethanolamine/10 mM EDTA (pH 7.4) was performed. Protein concentration was determined by absorbance at 280 nm (ε= 36,440 $M^{-1}cm^{-1}$) TbTIM and LmTIM. Enzymatic activity assays: Enzymatic activity was determined following the conversion of glyceraldehyde 3-phosphate into dihydroxyacetone phosphate. The decrease in absorbance at 340 nm was followed in a multi-cell Cary spectrophotometer at 25 ^{o}C. The reaction mixture (1 mL, pH 7.4) contained 100 mM triethanolamine, 10 mM EDTA, 0.2 mM NADH, 1 mM glyceraldehyde 3-phosphate and 0.9 units of _-glycerol phosphate dehydrogenase. The reaction was initiated by the addition of 5 ng/mL of the TbTIM. For the inhibition studies, TcTIM, LmTIM and TbTIM were incubated at a concentration of 5 mg/mL in a buffer containing 100 mM triethanolamine, 10 mM EDTA, pH 7.4 and 10% of DMSO at 36 _C. The mixture also contained the compounds at the indicated concentrations. Compounds were dissolved in DMSO. After 2 h, 10 µL was withdrawn and added to a final volume of 100 µL of the reaction mixture for the activity assay. The inhibition assay was performed in a 96-well microplate Varioskan spectrophotometer. None of the molecules tested here affected the activity of α-glycerol phosphate dehydrogenase, the enzyme used for trapping the product. The IC_{50} value was taken as the concentration of drug needed to reduce the enzymatic activity to 50%. The experiments were performed in triplicate.

Concentration of 796 (µM)	LmTIM inactivation (%)	TbTIM inactivation (%)
200	83.6	86.2
100	71.2	84
50	42.5	61
25	24.6	34
12.5	25.6	27
6	14.1	10
3	2.8	8

This section bibliography:

Aguilera, E., Varela, J., Birriel, E., Serna, E., Torres, S., Yaluff, G., de Bilbao, N.V.N.V., Aguirre-López, B., Cabrera, N., Díaz Mazariegos, S., de Gómez-Puyou, M.T.M.T., Gómez-Puyou, A., Pérez-Montfort, R., Minini, L., Merlino, A., Cerecetto, H., González, M., Alvarez, G., DeBilbao, N.V., Aguirre-López, B., Cabrera, N., DíazMazariegos, S., DeGómez-Puyou, M.T., Gómez-Puyou, A., Pérez-Montfort, R., Minini, L., Merlino, A., Cerecetto, H., González, M., Alvarez, G., de Bilbao, N.V.N.V., Aguirre-López, B., Cabrera, N., Díaz Mazariegos, S., de Gómez-Puyou, M.T.M.T., Gómez-Puyou, A., Pérez-Montfort, R., Minini, L., Merlino, A., Cerecetto, H., González, M., Alvarez, G., 2016. Potent and Selective Inhibitors of Trypanosoma cruzi Triosephosphate Isomerase with Concomitant Inhibition of Cruzipain: Inhibition of Parasite Growth through Multitarget Activity. ChemMedChem 11, 1328-1338. doi:10.1002/cmdc.201500385

Alvarez, G., Ma, P., Elena, C., Rivas, A., Cuchilla, K., Echeverr, G. a, Piro, O.E., Chorilli, M., Leal, S.M., Escobar, P., Serna, E., Torres, S., Yalu, G., Bilbao, N.I.V. De, Gonza, M., Cerecetto, H., Nacional, C., Cientí, D.I., Salvador, D. El, Salvador, S., Salvador, E., 2014. Optimization of Antitrypanosomatid Agents: Identi fi cation of Nonmutagenic Drug Candidates with in Vivo Activity. J. Med. Chem. 57, 3984-3999.

Álvarez, G., Perdomo, C., Coronel, C., Aguilera, E., Varela, J., Aparicio, G., Zolessi, F.R., Cabrera, N., Vega, C., Rolón, M., De Arias, A.R., Pérez-Montfort, R., Cerecetto, H., González, M., 2017. Multi-anti-parasitic activity of arylidene ketones and thiazolidene hydrazines against Trypanosoma cruzi and Leishmania spp. Molecules 22. doi:10.3390/molecules22050709

Álvarez, G., Varela, J., Cruces, E., Fernández, M., Gabay, M., Leal, S.M., Escobar, P., Sanabria, L., Serna, E., Torres, S., Figueredo Thiel, S.J., Yaluff, G., Vera de Bilbao, N.I., Cerecetto, H., González, M., 2015. Identification of a New Amide-Containing Thiazole as a Drug Candidate for Treatment of Chagas' Disease. Antimicrob. Agents Chemother. 59, 1398–1404. doi:10.1128/AAC.03814-14

Álvarez, G., Varela, J., Márquez, P., Gabay, M., Arias Rivas, C.E., Cuchilla, K., Echeverría, G.A., Piro, O.E., Chorilli, M., Leal, S.M., Escobar, P., Serna, E., Torres, S., Yaluff, G., Vera De Bilbao, N.I., González, M., Cerecetto, H., 2014. Optimization of antitrypanosomatid agents: Identification of nonmutagenic drug candidates with in vivo activity. J. Med. Chem. 57, 3984–3999. doi:10.1021/jm500018m

Ballell, L., Bates, R.H., Young, R.J., Alvarez-Gomez, D., Alvarez-Ruiz, E., Barroso, V., Blanco, D., Crespo, B., Escribano, J., González, R., Lozano, S., Huss, S., Santos-Villarejo, A., Martín-Plaza, J.J., Mendoza, A., Rebollo-Lopez, M.J., Remuiñan-Blanco, M., Lavandera, J.L., Pérez-Herran, E., Gamo-Benito, F.J., García-Bustos, J.F., Barros, D., Castro, J.P., Cammack, N., 2013. Fueling Open-Source Drug Discovery: 177 Small-Molecule Leads against Tuberculosis. ChemMedChem 8, 313–321. doi:10.1002/cmdc.201200428

Duffy, S., Sykes, M.L., Jones, A.J., Shelper, T.B., Simpson, M., Lang, R., Poulsen, S.A., Sleebs, B.E., Avery, V.M., 2017. Screening the medicines for malaria venture pathogen box across multiple pathogens reclassifies starting points for open-source drug discovery. Antimicrob. Agents Chemother. 61, 1–22. doi:10.1128/AAC.00379-17

Peña, I., Pilar Manzano, M., Cantizani, J., Kessler, A., Alonso-Padilla, J., Bardera, A.I., Alvarez, E., Colmenarejo, G., Cotillo, I., Roquero, I., De Dios-Anton, F., Barroso, V., Rodriguez, A., Gray, D.W., Navarro, M., Kumar, V., Sherstnev, A., Drewry,

D.H., Brown, J.R., Fiandor, J.M., Julio Martin, J., 2015. New compound sets identified from high throughput phenotypic screening against three kinetoplastid parasites: An open resource. Sci. Rep. 5. doi:10.1038/srep08771

Veale, C.G.L., Hoppe, H.C., 2018. Screening of the Pathogen Box reveals new starting points for anti-trypanosomal drug discovery. Medchemcomm 9, 2037–2044. doi:10.1039/c8md00319j

Vila, T., Lopez-ribot, J.L., 2017. Screening the Pathogen Box for Identification of Candida albicans Biofilm Inhibitors. Antimicrob Agents Chemother 61, 1–9. doi:10.1128/AAC.02006-16
